# Sequence Variations of Latent Membrane Protein 2A in Epstein-Barr Virus-Associated Gastric Carcinomas from Guangzhou, Southern China

**DOI:** 10.1371/journal.pone.0034276

**Published:** 2012-03-28

**Authors:** Jing Han, Jian-ning Chen, Zhi-gang Zhang, Hai-gang Li, Yun-gang Ding, Hong Du, Chun-kui Shao

**Affiliations:** 1 Department of Pathology, The Third Affiliated Hospital, Sun Yat-sen University, Guangzhou, Guangdong Province, China; 2 Department of Pathology, The Second Affiliated Hospital, Sun Yat-sen University, Guangzhou, Guangdong Province, China; 3 Department of Pathology, Guangzhou First Municipal People's Hospital, Guangzhou, Guangdong Province, China; Karolinska Institutet, Sweden

## Abstract

Latent membrane protein 2A (LMP2A), expressed in most Epstein-Barr virus (EBV)-associated malignancies, has been demonstrated to be responsible for the maintenance of latent infection and epithelial cell transformation. Besides, it could also act as the target for a CTL-based therapy for EBV-associated malignancies. In the present study, sequence variations of LMP2A in EBV-associated gastric carcinoma (EBVaGC) and healthy EBV carriers from Guangzhou, southern China, where nasopharyngeal carcinoma (NPC) is endemic, were investigated. Widespread sequence variations in the LMP2A gene were found, with no sequence identical to the B95.8 prototype. No consistent mutation was detected in all isolates. The immunoreceptor tyrosine-based activation motif (ITAM) and PY motifs in the amino terminus of LMP2A were strictly conserved, suggesting their important roles in virus infection; while 8 of the 17 identified CTL epitopes in the transmembrane region of LMP2A were affected by at least one point mutation, which may implicate that the effect of LMP2A polymorphisms should be considered when LMP2A-targeted immunotherapy is conducted. The polymorphisms of LMP2A in EBVaGC in gastric remnant carcinoma (GRC) were for the first time investigated in the world. The LMP2A sequence variations in EBVaGC in GRC were somewhat different from those in EBVaGC in conventional gastric carcinoma. The sequence variations of LMP2A in EBVaGC were similar to those in throat washing of healthy EBV carriers, indicating that these variations are due to geographic-associated polymorphisms rather than EBVaGC-associated mutations. This, to our best knowledge, is the first detailed investigation of LMP2A polymorphisms in EBVaGC in Guangzhou, southern China, where NPC is endemic.

## Introduction

Epstein-Barr virus (EBV) is a ubiquitous herpes virus that was originally identified in a human Burkitt lymphoma cell line [1]. It is implicated in the etiology of human lymphoid neoplasms including Burkitt lymphoma, Hodgkin lymphoma, and B-cell lymphomas among immunosuppressed patients [2]. EBV has also been suspected to cause epithelial malignancies such as nasopharyngeal carcinoma (NPC) and a subset of gastric carcinoma which is defined as EBV-associated gastric carcinoma (EBVaGC) [2]. EBVaGC represents about 10% of gastric carcinoma worldwide; however, the frequency varies from country to country and ranges from 1.3% to 20.1% [3], [4], [5]. The first large study (>500 cases) was conducted in Japan, in which EBVaGC accounted for 6.9% of the cases (67 of 970 cases) [6]. Besides Japan, van Beek et al. [7] detected 41 (7.2%) EBVaGCs out of 566 gastric carcinoma cases in the Netherlands. In our preliminary study, we found that the proportion of EBVaGC in gastric carcinoma in Guangzhou, southern China was 6.7% (45/676). According to a recent meta-analysis, the pooled estimate of frequency of EBVaGC in gastric carcinoma in America, Europe and Asia was 9.9%, 9.2% and 8.3%, respectively [8].

After infection, EBV persists in host in latency cycle and constitutively expresses a limited set of viral gene products, the so-called latent products, which comprise six EBV nuclear antigens (EBNAs 1, 2, 3A, 3B, 3C and -LP), three latent membrane proteins (LMPs 1, 2A and 2B), two EBV-encoded small non-coding RNAs (EBERs 1 and 2) and the BamHI A rightward transcripts (BARTs) [9]. Three latency patterns have been described depending on which of these latent products are expressed. Latency I is limited to only EBERs, BARTs and EBNA1 expression; latency II includes LMP1 and LMP2 in addition; and latency III is defined by expression of EBERs, BARTs, all six EBNA proteins and three LMP proteins [9]. EBVaGC belongs to latency I. However, zur Hausen *et al.* [10], Sugiura *et al.* [11] and Luo *et al.* [12] examined the viral gene expression in EBVaGCs at the mRNA level and found that LMP2A mRNA was detected in 7 of 9 (77.8%), 3 of 7 (42.9%) and 4 of 11 (36.4%) cases, respectively. In addition, zur Hausen *et al.* [10] also found that BARF1 mRNA were expressed in 9 of 9 (100%) EBVaGC cases.

LMP2A consisting of 497 amino acids (aa) is encoded by LMP2 gene, whose mRNA containing 9 exons [13]. LMP2A includes a 119 aa cytoplasmic amino-terminal domain, 12 hydrophobic transmembrane domains and a 27 aa cytoplasmic carboxyl-terminal domain [14]. The amino-terminal cytoplasmic domain of LMP2A, encoded by exon 1, contains eight tyrosine (Tyr, Y) residues, two of which (Tyr74 and Tyr85) are involved in the formation of an immunoreceptor tyrosine-based activation motif (ITAM) and play a crucial role in its function [15]. This motif acts as a negative regulator of Src family and blocks B-cell receptor (BCR)-mediated signal transduction [13], [16]. A membrane proximal tyrosine residue (Tyr112) binds the Lyn tyrosine kinase and mediates the constitutive phosphorylation of the other tyrosine residues in LMP2A [15]. LMP2A also recruits Nedd4-like ubiquitin protein ligases to promote the degradation of Lyn and LMP2A by an ubiquitin-dependent mechanism through phosphotyrosine (PY) motifs [16], [17]. It has been proved that Tyr112 mutation could reduce both the degree and duration of phosphorylation of key components of the BCR signaling cascade [18].

Besides its function on B cells, LMP2A can also act on epithelial cells. LMP2A induces an epithelial-mesenchymal transition and increases the number of side population stem-like cancer cells in NPC [19]. LMP2A is reported to transform epithelial cells, affect cell growth and differentiation pathways in a human keratinocyte cell line (HaCaT) [20]. LMP2A is also demonstrated to inhibit transforming growth factor-β1-induced apoptosis in a gastric carcinoma cell line HSC-39, through activation of the phosphatidylinositol-3-kinase (PI3K)-Akt pathway [21]. Moreover, recently, it is demonstrated that LMP2A up-regulates survivin gene expression through the nuclear factor-kappa B (NF-κB) pathway in EBVaGC [22]. In addition, LMP2A up-regulates cellular DNA methyltransferase 1 (DNMT1) through the phosphorylation of STAT3, causing promoter hypermethylation of a tumor suppressor gene, PTEN, in EBVaGC [23]. Based on these observations, it is conceivable that any aa change in LMP2A sequence may lead to malfunctions of LMP2A.

LMP2A at least possesses 17 target epitopes of cytotoxic T lymphocytes (CTLs) [24], [25], [26], [27], [28]. CTLs are very important in controlling the long-term persistence of EBV infection; disruption of the balance between CTL and virus-infected B cells result in EBV-associated disease [29]. Thus, LMP2A could act as a potential target for a CTL-based therapy for EBV-associated malignancies [24], [30], [31]. Studies are very encouraging and suggest that a CTL-based therapy may be of benefit in treating EBV-associated malignancies [32], [33]. However, according to a recently published study [14], 9 of 16 CTL epitopes in LMP2A were affected by at least one point mutation. These epitope mutations may result in a reduced CTL response and confer complexity to proposed immunotherapeutic approaches for EBV associated malignancies.

The sequence variations of LMP2A in EBVaGC have been explored in only two reports [14], [29]. The first one is from Japan. Tanaka *et al.* [29] determined the complete sequence of LMP2A in three EBVaGC cases. In addition, the sequences of exons 2, 6 and 7 of LMP2A were determined in another one, three and two EBVaGC cases, respectively. They found eight aa substitutions, three of which (Y23D in exon 1, S348T in exon 6 and S444T in exon 7) were identified in almost all cases tested. Of these three, S348T and S444T were located within HLA A11- and A25-restricted CTL epitopes, respectively. The other one is from northern China. Wang *et al.* [14] analyzed the sequence variations of exon 1 and exons 2–8 of LMP2A in 40 and 30 EBVaGC cases, respectively. Thirty aa substitutions were found and 9 of 16 CTL epitopes in LMP2A were affected by at least one point mutation.

Guangzhou, located in southern China, is well known as the high-incidence area of NPC in the world [34]. NPC is known as an EBV-associated epithelial malignancy. In addition, a special EBV variant, variant-type “f”, is predominant in NPC and is strongly associated with NPC in Guangzhou [35], [36]. In our previous study [37], we found that the predominant EBV variant in EBVaGC in Guangzhou was prototype F, which is different from that in NPC in this area. Moreover, a new identified variant, mut-W1/I1 variant, which shows a T to C mutation at position 148,972 (wild type EBV coordinates), was found in the majority of the EBVaGCs in Guangzhou. However, this mutation could not be found in the NPC-derived EBV stain GD1 [38]. These provide evidence that there may be a disease-related association between EBV variants, at least in EBVaGC versus NPC in patients drawn from the same population. Thus, the sequence variations of LMP2A in EBVaGC in Guangzhou were of our interest. It may be of help in clarifying the pathogenic roles of EBV in epithelial malignancies to compare the LMP2A variations in EBVaGC in Guangzhou with those in NPC and in healthy EBV carrier in the same area and those in EBVaGC in other areas.

Gastric remnant carcinoma (GRC) is defined as a carcinoma occurring in the gastric stump at least 5 years after surgery for benign diseases such as gastric ulcer and duodenal ulcer [39], [40]. In our preliminary study, we found that the proportion of EBVaGC in GRC was significantly higher than that in conventional gastric carcinoma (CGC) which occurs in the intact stomach in Guangzhou (30.8% vs. 6.7%). Similar findings were also reported by other groups in Japan (27.1% vs. 6.4%) [41], Korea (29% vs. 6%) [42] and Netherlands (35% vs. 8%) [43]. EBV seems more aggressive in EBVaGC in GRC than that in CGC. However, our previous study has showed that the EBV genome polymorphisms at the EBNA-3C region, BamHI-F region, BamHI-W1/I1 boundary region, and exons 1 & 3 of the LMP1 gene in EBVaGC in GRC were similar to those in EBVaGC in CGC [44]. Are the LMP2A sequence variations in EBVaGC in GRC different from those in EBVaGC in CGC? To date, no study on polymorphisms of LMP2A in EBVaGC in GRC is available.

In the present study, the sequence variations of LMP2A in EBVaGC in CGC, EBVaGC in GRC and throat washing (TW) samples of healthy EBV carriers in Guangzhou, southern China, where NPC is endemic, were analyzed. We are trying to explore the association between sequence variations of LMP2A and EBVaGC, as well as the possibility of a CTL-based therapy for EBVaGC.

## Materials and Methods

### Ethics Statement

This study was approved by the Clinical Research Ethics Committee of the Third Affiliated Hospital, Sun Yat-sen University. Written informed consents were taken from all the patients and healthy donors and ethical guidelines under Declaration of Helsinki were followed.

### Subjects

Six hundred and seventy-six CGC cases and twenty-six GRC cases were collected from the Second and Third Affiliated Hospitals of Sun Yat-sen University and the Guangzhou First Municipal People's Hospital, Guangzhou, China, from January 1, 2000 to December 31, 2006. All the tumor specimens were from surgical resection cases. Paraffin blocks and clinicopathologic data, which include age and sex of the patient, the location, histological type, invasion depth and lymphatic and hematogenous metastases of the tumor, were obtained from the archives of the three Departments of Pathology. Among the 676 CGC cases, 53 cases were early gastric carcinomas, while the remaining 623 cases were advanced gastric carcinomas. All GRC cases were advanced gastric carcinomas.

All GRC cases had received partial gastrectomy for reasons of benign diseases, including gastric ulcer (16 cases), duodenal ulcer (7 cases), combined gastric ulcer and duodenal ulcer (2 cases) and pyloric stenosis (1 case). Cases with GRC after gastrectomy for gastric cancer were excluded in this study.

Histology of the gastric carcinomas was classified as intestinal- and diffuse-type according to the Lauren classification [45]. The tumor location and depth of invasion were classified according to the Japanese classification [46].

TW samples from 50 healthy donors were also collected in the same geographical regions by gargling with 15 ml of phosphate buffered saline (PBS). Polymerase chain reaction (PCR) with the *Bam*HI K region primers was used to screen the TWs for EBV genome. Seventeen (34%) of the 50 TW samples were positive for the EBV *Bam*HI K fragment.

All the patients and healthy donors were inhabitants in Guangdong province with local dialects.

### Construction of tumor tissue microarrays

The tumor tissue microarray (TMA) blocks were constructed as described by Kononen et al. [47]. A 12×8 cylinder matrix was defined on a 3.5×2.5 cm^2^ paraffin wax block, and position-specific blank cylinders were adopted for orientation during microscopy analysis. A total of 14 TMA blocks were constructed, where each TMA block contained up to 94 tissue cores from 47 cases of advanced gastric carcinoma and each case was composed of 2 tissue cores. An adequate case was defined by tumor occupancy of more than 10% of the core area.

### *In situ* hybridization

An *in situ* hybridization (ISH) assay was performed on the TMA sections, as well as the donor block sections of the positive tissues on the TMA sections, and on the block sections of early gastric carcinoma cases with a commercially available EBV oligonucleotide probe complementary to the EBV-encoded small RNA-1 (EBER-1) (PanPath, Amsterdam, Netherlands), according to the manufacturer's instructions. The hybridization signals were visualized with 3,3′-diaminobenzidine (DAB; Vector Laboratories, Inc.), and positive signals were recognized as dark brown nuclear staining under light microscopy. Sections from a known EBER-1-positive NPC tissue were used as the positive control and a sense probe for EBER-1 was used as the negative control.

### Immunostaining for LMP2A

The immunostaining for LMP2A was performed using the two-step EnVision immunohistochemical procedure (Dako, Denmark) as previously described [44]. The monoclonal antibody against LMP2A (clone 4A11B3A3; a gift from Prof. Mu-sheng Zeng) [19] was applied. The signals were visualized with DAB, and the slides were counterstained with Mayer's hematoxylin. The LMP2A-positive NPC tissues were used as positive controls. PBS other than the primary antibodies was used as the negative control. Tumors were considered positive if 10% or more of the neoplastic cells were stained.

### DNA extraction

Total DNA was extracted from EBV positive paraffin sections and TW samples of healthy donors using the NucleoSpin Tissue Kit (MACHEREY-NAGEL GmbH & Co. KG, Düren, Germany) according to the manufacturer's instructions. Paraffin blocks without any samples were used as negative controls throughout the procedures. EBV-positive cell line B95.8 was used as positive control. The extracted DNA sample was dissolved in 100 µl of TE buffer.

### Polymerase chain reaction

PCR was performed with 2 µl of DNA in a 50 µl total reaction mixture containing 10 mM Tris-HCl (pH 8.0), 50 mM KCl, 1.5 mM MgCl_2_, 200 µM dNTP, 0.5 µM of each primer and 1.25 U *Taq* Polymerase (TaKaRa, Dalian, China). EBV *Bam*HI K region and exons 1–8 of LMP2A were amplified. Exon 9 of LMP2A was not amplified in the present study, because neither functional domain nor sequence variation was detected in this fragment [29]. The primers are listed in Table 1. The amplification protocol was one cycle at 95°C for 5 min, followed by 40 cycles of denaturation at 95°C for 1 min, annealing at 55°C for 1 min and elongation at 72°C for 1 min, and a final extension at 72°C for 10 min. The amplified products were detected on 2% agarose gels stained with 0.5 µg/ml of ethidium bromide and photographed under UV light.

**Table 1:** Primers used in the present study.

Transcript	Product Size	Sequence (5′-3′)	B95.8 Genomic Coodinates	Reference
*Bam*HI K	270 bp	5′ primer GTCATCATCATCCGGGTCTC	109110–109129	
		3′ primer TTCGGGTTGGAACCTCCTTG	109379–109360	
Exon 1a*	279 bp	5′ primer CGGTTTCAGCATCACAGG	166451–166468	
		3′ primer GGGGCTCTTCATTAGATTCACG	166729–166708	
Exon 1b*	327 bp	5′ primer CCATCTGCTTCTGGCTCTT	166654–166672	
		3′ primer CGCCCTTATTATTGATGTGA	166980–166961	
Exon 2	292 bp	5′ primer TTGCTCTATTCACCCTTACT	11–30	[29]
		3′ primer ATGCATTGTAAATGGTGCGT	302–283	
Exon 3	159 bp	5′ primer ACAGTTCACTTCCTCTGCTT	330–349	[29]
		3′ primer ACCTGCATTTCATAACAGAG	488–469	
Exon 4	309 bp	5′ primer GGCATGACGTCAACTTTACT	510–529	[29]
		3′ primer AAGGCAAGCAATGTCACACG	818–799	
Exon 5	141 bp	5′ primer CAGGACTAACCATGCCATCT	841–860	[29]
		3′ primer AGTAATGAGTTAAAGGAAGG	981–962	
Exon 6	283 bp	5′ primer AATCGCAGCTCTAACTTGGC	993–1012	
		3′ primer GGAACAAAGCGTAAAATGTGTG	1275–1254	
Exon 7	285 bp	5′ primer CTATTGGATTGTAACACACA	1240–1259	[29]
		3′ primer AAGGCAACACCTGTTGGTGT	1524–1505	
Exon 8	177 bp	5′ primer ACATGACTTACATGGGTTTG	1544–1563	[29]
		3′ primer TACACGTGATAGTGTCTCTA	1720–1701	

* Exon 1 was amplified as two fragments because the whole fragment was too long for DNA amplification in paraffin-embedded tissue. Exon 1a and exon 1b were overlapped so they together covered the whole exon 1 fragment.

### DNA sequencing

The products of the PCR reaction were extracted and purified using QIAquick PCR Purification Kit (QIAGEN, Hilden, Germany). The purified PCR products were then subjected to DNA sequencing. Cycle sequencing was carried out using the ABI PRISM BigDye Terminator Cycle Sequencing Ready Reaction Kit (PE Applied Biosystems, CA, USA) according to the manufacturer's manual. Sequence analysis was performed on the 3730xl DNA Analyzers and the data were analyzed with Seq Ed. software (PE Applied Biosystems). All sequences were performed uni-directionally. The results were then compared with the sequences of EBV strains B95.8 (GenBank Accession No.: V01555) and GD1 (GenBank Accession No.: AY961628).

### Statistical analysis

Fisher's exact test or Student's *t* test was used for statistical analysis in the present study. The results were considered to be statistically significant at a *p*-value of less than 0.05. All the *p*-values presented in the present study are two-sided.

## Results

### EBER-1 expressed in 6.7% CGCs and 30.8% GRCs

**Figure 1:**
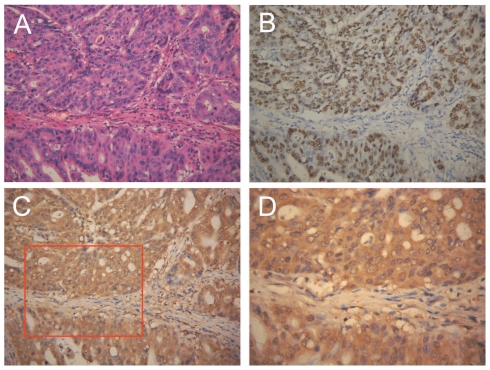
Expression of LMP2A in EBVaGC. (A) *H&E* staining of a moderate differentiated tubular gastric adenocarcinoma. The histology of this case was intestinal-type carcinoma according to the Lauren classification. *(Original magnification ×200)*. (B) EBER-1 *in situ* hybridization of the same case. The EBER-1-positive signals were restricted only to the tumor nuclei but not in surrounding non-tumor cells. *(Original magnification ×200)*. (C) Immunohistochemistry staining for LMP2A of the same case. Positive staining can be seen in the tumor cells but not in infiltrating lymphocytes in and around the tumor nests. *(Original magnification ×200)*. (D) Higher magnification of panel C. Positive signals of LMP2A were labeled in the cytoplasm and membrane of the tumor cells. *(Original magnification ×400)*.

Forty-five (6.7%) out of the 676 CGC cases and 8 (30.8%) out of the 26 GRC cases displayed EBER-1 ISH positive signals (Figure 1A,B) and were considered EBVaGCs. In most EBVaGC cases, the positive signals were restricted only to the nuclei of carcinoma cells. However, in three EBVaGC cases in CGC, some dysplastic epithelial cells adjacent to the carcinoma also showed EBER-positive signals, while the lymphocytes infiltrating in and around the carcinoma tissues showed negative signals.

The clinicopathologic features of EBVaGC in CGC and GRC are shown in Table 2.

**Table 2:** Clinicopathologic characteristics of EBVaGC in CGC and GRC.

Variables	EBVaGC in CGC (n)	EBVaGC in GRC (n)	*p* *
Gender			
Male	37	8	0.333
Female	8	0	
Age (years)			
≤40	11	0	0.181
>40	34	8	
Range	23∼76	55∼79	
Mean ± SD†	51.5±13.9	67.6±7.4	0.000
Location‡			
Cardia	15	/	
Body	14	/	
Antrum	14	/	
Whole§	2	/	
Stump	/	8	
Histology∥			
Intestinal	8	0	0.333
Diffuse	37	8	
Invasion‡			
T1	0	0	1.000
T2	4	0	
T3	35	7	
T4	6	1	
Stage (pTNM)‡			
1a	0	0	0.442
1b	1	0	
2	8	3	
3a	15	4	
3b	9	0	
4	12	1	

* *p*-values were obtained from Fisher's exact tests or Student's *t* tests.

† SD: standard deviation.

‡ Japanese classification.

§ Cases involved the whole stomach.

∥ Lauren classification.

### LMP2A expressed in 53.3% EBVaGCs in CGC and 62.5% EBVaGCs in GRC

The immunohistochemistry of LMP2A was performed in all 53 EBVaGC cases. Twenty-four (53.3%) of the 45 EBVaGC cases in CGC and 5 (62.5%) of 8 EBVaGC cases in GRC showed diffuse positive signals in the membrane and cytoplasm of the tumor cells (Figure 1C,D).

There was no correlation between LMP2A expression and the clinicopathologic parameters of EBVaGC, which include the age and sex of the patient, the location, histology and stage of the tumor (all p>0.05, data not shown).

### Sequence variations of LMP2A exon 1 in EBVaGC in CGC, EBVaGC in GRC and TW of healthy EBV carriers

LMP2A exon 1 was successfully amplified in 45 EBVaGCs in CGC, 8 EBVaGCs in GRC and 17 TWs of healthy EBV carriers. All sequences were compared with the B95.8 prototype sequence. Six loci of nucleotide substitution were identified, 4 of which result in aa changes. Among them, the nucleotide mutation (t→g) at position 166627, which results in a Y→D substitution at aa 23, was detected in 35 (77.8%) EBVaGCs in CGC, 8 (100%) EBVaGCs in GRC and 17 (100%) TWs of healthy EBV carriers. The distribution of nucleotide variations of LMP2A exon 1 in EBVaGC in CGC, EBVaGC in GRC and TW of healthy EBV carriers was shown in Table 3.

**Table 3:** Distribution of nucleotide variations of LMP2A exon 1 in EBVaGC in CGC, EBVaGC in GRC and TW of healthy EBV carriers.

	Nucleotide	Amino acid	No. of isolates (%)		
			EBVaGCin CGC(n = 45)	EBVaGCin GRC(n = 8)	TW of healthy EBV carriers(n = 17)
Exon 1	166604 g→a*	15 S→N	1(2.2)	0(0)	4(23.5)
	166627 t→g*	23 Y→D	35(77.8)	8(100.0)	17(100.0)
	166796 c→a*	79 T→N	3(6.7)	0(0)	0(0)
	166805 a→c*	82 Q→P	3(6.7)	0(0)	0(0)
	166810 c→t	84 L→L	3(6.7)	0(0)	0(0)
	166896 c→t	112 Y→Y	15(33.3)	0(0)	0(0)

* missense mutation.

### Sequence variations of LMP2A exons 2–8 in EBVaGC in CGC,EBVaGC in GRC and TW of healthy EBV carriers

DNA sequences of LMP2A exons 2–8 were obtained from 36 EBVaGCs in CGC, 8 EBVaGCs in GRC and 17 TWs of healthy EBV carriers, respectively. In total, 39 nucleotide mutations were found, which cause 18 aa changes. Among them, 12 loci of aa mutation (codons 153, 169, 171, 208, 248, 254, 255, 350, 370, 391, 426 and 444) were detected in >40% isolates from both EBVaGCs in CGC and TWs of healthy EBV carriers. The distribution of these mutations between EBVaGCs in CGC and TWs of healthy EBV carriers did not differ significantly (*p*>0.05). In EBVaGCs in GRC, aa mutations at codons 153, 169, 171, 208, 370, 391, 426 and 444 were found in >40% cases, while at codon 350, the I to V substitution was detected in 37.5% of the cases. The distribution of these mutations between EBVaGCs in CGC and EBVaGCs in GRC did not differ significantly, either (*p*>0.05). At codons 248, 254 and 255, no aa mutation was detected in EBVaGCs in GRC. The difference of these mutation between EBVaGCs in GRC and EBVaGCs in CGC was statistically significant (*p* = 0.014). At aa 348, 6 (16.7%) EBVaGCs in CGC, 4 (50%) EBVaGCs in GRC and 9 (52.9%) TWs of healthy EBV carriers harbored an S to T substitution. The difference of this mutation between EBVaGCs in CGC and TWs of healthy EBV carriers was statistically significant (*p* = 0.010). However, the distributions of this mutation between EBVaGCs in CGC and EBVaGCs in GRC as well as between EBVaGCs in GRC and TWs of healthy EBV carriers did not differ significantly (*p* = 0.064 and 1.000, respectively). The sequences of exons 5 and 8 were identical with those of B95.8, that is, no variation was detected in these two fragments. The distribution of nucleotide variations of LMP2A exons 2–8 in EBVaGC in CGC, EBVaGC in GRC and TW of healthy EBV carriers was shown in Table 4.

**Table 4:** Distribution of nucleotide variations of LMP2A exons 2–8 in EBVaGC in CGC, EBVaGC in GRC and TW of healthy EBV carriers.

	Nucleotide	Amino acid	No. of isolates (%)		
			EBVaGCin CGC(n = 36)	EBVaGCin GRC(n = 8)	TW of healthy EBV carriers(n = 17)
Exon 2	100 c→g	133 L→L	28(77.8)	6(75)	17(100%)
	133 c→t	144 F→F	0(0)	0(0)	1(5.9)
	157 g→c	152 V→V	19(52.8)	4(50)	8(47.1)
	158 a→c*	153 T→P	1(2.8)	0(0)	0(0)
	159 c→g*	153 T→S	19(52.8)	4(50)	8(47.1)
	185 a→c	162 L→L	1(2.8)	0(0)	0(0)
	196 a→c	165 A→A	1(2.8)	0(0)	0(0)
	207 g→a*	169 S→N	21(58.3)	7(87.5)	9(52.9)
	213 a→c*	171 Y→S	19(52.8)	4(50)	8(47.1)
	247 g→t	182 V→V	19(52.8)	4(50)	8(47.1)
	250 a→c	183 L→L	1(2.8)	0(0)	0(0)
	253 g→t	184 V→V	25(69.4)	7(87.5)	17(100)
Exon 3	391 g→a	201 E→E	28(77.8)	6(75)	9(52.9)
	410 c→a*	208 L→I	28(77.8)	6(75)	9(52.9)
Exon 4	571 g→a	234 A→A	20(55.6)	2(25)	10(58.8)
	579 g→c*	237 R→T	1(2.8)	0(0)	0(0)
	599 g→c*	244 V→L	1(2.8)	0(0)	0(0)
	613 c→g*	248 I→M	18(50)	0(0)	7(41.2)
	629 g→c*	254 V→L	18(50)	0(0)	7(41.2)
	632 c→g*	255 L→V	18(50)	0(0)	7(41.2)
Exon 6	1068 g→c*	348 S→T	6(16.7)	4(50)	9(52.9)
	1073 c→g*	350 I→V	17(47.2)	3(37.5)	7(41.2)
	1075 c→t*		17(47.2)	3(37.5)	7(41.2)
	1105 c→a	360 L→L	19(52.8)	5(62.5)	9(52.9)
	1134 t→c*	370 I→T	19(52.8)	5(62.5)	9(52.9)
	1138 t→c	371 A→A	17(47.2)	3(37.5)	7(41.2)
	1177 c→t	384 S→S	17(47.2)	3(33.3)	7(41.2)
	1182 c→g*	386 T→S	1(2.8)	0(0)	0(0)
	1195 c→t	390 P→P	17(47.2)	3(37.5)	7(41.2)
	1196 a→c*	391 S→R	19(52.8)	5(62.5)	9(52.9)
Exon 7	1302 g→c	398 L→L	0(0)	1(12.5)	0(0)
	1323 c→t	405 F→F	16(44.4)	3(37.5)	11(64.7)
	1350 c→a	414 G→G	23(63.9)	4(50)	13(76.5)
	1374 a→c	422 P→P	24(66.7)	6(75)	13(76.5)
	1385 g→c*	426 C→S	23(63.9)	4(50)	13(76.5)
	1389 c→t	427 L→L	2(5.6)	0(0)	0(0)
	1392 t→c	428 G→G	23(63.9)	4(50)	13(76.5)
	1435 a→g*	443 M→V	1(2.8)	0(0)	0(0)
	1438 t→a*	444 S→T	29(80.6)	7(87.5)	15(88.2)

No nucleotide variation was detected in exons 5 and 8 of LMP2A.

* missense mutation.

### ITAM, PY motifs in the amino terminus of LMP2A were strictly conserved

Several functional critical motifs, including ITAM, PY motifs, were identified in the amino terminus of LMP2A. Their sequence polymorphisms were analyzed in the present study. The ITAM, consisting of paired pivotal tyrosines and leucines (YxxL) at Y74 and Y85, was conserved in all isolates. The two PY motifs (PPPPY Y60 and Y101), were not affected by mutations in any isolates, either.

### Eight out of seventeen CTL epitopes showed variations

In the present study, 8 of the 17 identified CTL epitopes were affected by at least one point mutation. The distributions of aa variations of CTL epitopes in EBVaGC in CGC, EBVaGC in GRC and TW of healthy EBV carriers were summarized in Table 5. Among the mutant CTL epitopes, the HLA-A11-restricted CTL epitope (codons 340 to 350) identified two mutations: S348T and I350V. The first mutation was detected in 16.7% (6/36) of EBVaGCs in CGC, 50% (4/8) of EBVaGCs in GRC and 52.9% (9/17) of TWs of healthy EBV carriers, while the second mutation was detected in 47.2% (17/36) of EBVaGCs in CGC, 37.5% (3/8) of EBVaGCs in GRC and 41.2% (7/17) of TWs of healthy EBV carriers. The distribution of these mutations between EBVaGCs in CGC and TWs of healthy EBV carriers was statistically significantly different (*p* = 0.009), while that between EBVaGCs in CGC and EBVaGCs in GRC and that between EBVaGCs in GRC and TWs of healthy EBV carriers were not statistically significantly different (*p* = 0.121 and 1.000, respectively).

**Table 5:** Amino acid variations of CTL epitopes in EBVaGC in CGC, EBVaGC in GRC and TW of healthy EBV carriers.

HLA restriction	LMP2A residues	Epitope sequence														No. of isolates (%)		
																EBVaGC in CGC (n = 36)	EBVaGC in GRC (n = 8)	TW of healthy EBV carriers(n = 17)
ND*	141–154	A	S	C	F	T	A	S	V	S	T	V	V	T	A	16(44.4)	4(50)	9(52.9)
		–	–	–	–	–	–	–	–	–	–	–	–	P	–	1(2.8)	0(0)	0(0)
		–	–	–	–	–	–	–	–	–	–	–	–	S	–	19(52.8)	4(50)	8(47.1)
B60	200–208	I	E	D	P	P	F	N	S	L						8(22.2)	2(25)	8(47.1)
		–	–	–	–	–	–	–	–	I						28(77.8)	6(75)	9(52.9)
B27	236–244	R	R	R	W	R	R	L	T	V						35(97.2)	8(100)	17(100)
		–	T	–	–	–	–	–	–	L						1(2.8)	0(0)	0(0)
ND*	249–262	M	F	L	A	C	V	L	V	L	I	V	D	A	V	18(50)	8(100)	10(58.8)
		–	–	–	–	–	L	V	–	–	–	–	–	–	–	18(50)	0(0)	7(41.2)
A11	340–350	S	S	C	S	S	C	P	L	S	K	I				13(36.1)	1(12.5)	1(5.9)
		–	–	–	–	–	–	–	–	T	–	–				6(16.7)	4(50)	9(52.9)
		–	–	–	–	–	–	–	–	–	–	V				17(47.2)	3(37.5)	7(41.2)
A24	419–427	T	Y	G	P	V	F	M	C	L						13(36.1)	4(50)	4(23.8)
		–	–	–	–	–	–	–	S	–						23(63.9)	4(50)	13(76.5)
A2	426–434	C	L	G	G	L	L	T	M	V						13(36.1)	4(50)	4(23.8)
		S	–	–	–	–	–	–	–	–						23(63.9)	4(50)	13(76.5)
A25	442–451	V	M	S	N	T	L	I	S	A	W					6(16.7)	1(12.5)	2(11.8)
		–	V	–	–	–	–	–	–	–	–					1(2.8)	0(0)	0(0)
		–	–	T	–	–	–	–	–	–	–					29(80.6)	7(87.5)	15(88.2)

Sequences listed are epitope sequences of the B95.8 prototype. Only the mutant amino acids are shown; “–” indicates sequence identical to B95.8.

* ND: Not determined.

## Discussion

The present study not only described the expression of LMP2A in EBVaGC but also for the first time revealed the polymorphisms of LMP2A in EBVaGC in Guangzhou, southern China, which is an endemic area of NPC. Widespread sequence variations in the LMP2A gene were found, with no sequence identical to the B95.8 prototype. No consistent mutation was detected in all isolates. The ITAM and PY motifs in the amino terminus of LMP2A were strictly conserved, while 8 of the 17 identified CTL epitopes were affected by at least one point mutation.

In the present study, LMP2A was detected in 53.3% (24/45) of the EBVaGC cases in CGC and 62.5% (5/8) of the EBVaGC cases in GRC by immunohistochemistry (IHC). zur Hausen *et al.* [10], Sugiura *et al.* [11] and Luo *et al.* [12] examined the viral gene expression in EBVaGCs at the mRNA level and found that LMP2A mRNA was detected in 7 of 9 (77.8%), 3 of 7 (42.9%) and 4 of 11 (36.4%) cases, respectively. The different detection rates of LMP2A in EBVaGC may relate to the sentitivity of detection methods used. Using reverse transcription-PCR (RT-PCR), LMP2A mRNA was detected in 14 of 27 (51.9%) EBVaGC cases in total. By IHC, LMP2A expression was found in 29 of 53 (54.7%) EBVaGC cases in total. The detection rates of LMP2A in EBVaGC by RT-PCR and IHC are similar. These findings indicate that LMP2A is expressed in about half of the EBVaGC cases, irrespective of the detection methods used. Thus, EBVaGC could be divided into two subgroup based on the LMP2A expression. The first subgroup of EBVaGC does not express LMP2A, whose EBV latency pattern is the same with that of Burkitt lymphoma (latency I) [48]. The second subgroup of EBVaGC expresses LMP2A; thus, its EBV latency pattern is somewhat different from the conventional latency I represented by Burkitt lymphoma [48], and may represent a novel EBV latency pattern. Since LMP2A has been demonstrated to play important roles in the oncogenic processes in EBVaGC [22], [23], and about half of the EBVaGCs express LMP2A, we suggest designating the EBV latency pattern in EBVaGC as latency Ia and Ib, based on the absence and presence of LMP2A expression, respectively.

In the amino terminus of LMP2A, six nucleotide mutations and four resultant aa changes were found in EBVaGC in CGC. These four loci of aa substitution were also found in EBVaGC in northern China [14]. However, except for aa 23, the other three loci were not detected in 3 EBVaGCs in Japan [29]. Y23 is one of the eight tyrosine residues located in the amino terminus of LMP2A. The Y to D substitution at aa 23 was detected in 77.8% (35/45) EBVaGCs in CGC, 100% (8/8) EBVaGCs in GRC and 100% (17/17) TWs of healthy EBV carriers in the present study. It was also detected in all isolates from EBVaGC in Japan (3/3) [29] and in northern China (40/40) [14]. In addition, 100% (36/36) NPCs and 100% (40/40) TWs of healthy EBV carriers from northern China harbored this mutation, too [14]. Moreover, Y23D was also detected in all isolates from 8 NPCs (one from Egypt, one from continental USA, one from Alaska, three from China, and two from Malaysia), 4 post-transplant lymphomas from the Unite States, 2 Burkitt lymphomas (one from Central Africa and one from continental USA/Mediterranean Africa), irrespective of either disease type or geographical origin [49]. Furthermore, the tyrosine (Y23) is not found in the LMP2A-like protein sequence of herpesvirus papio from baboons [50]. Therefore, the amino acid substitution (Y→D) at codon 23 might not affect LMP2A functions. Aside from this substitution, the other seven tyrosine residues (Y31, Y60, Y64, Y74, Y85, Y101, and Y112) were all conserved in all the isolates. Thus, the ITAM and PY motifs were strictly conserved in EBVaGC and TW of healthy EBV carriers, indicating their critical role in the maintenance of gene functions.

In the transmembrane region (exons 2–8) of LMP2A, 39 nucleotide mutations were found, which cause 18 aa changes. Except for T153P and M443V, the other 16 aa substitutions were also detected in EBVaGC in northern China [14]. As we know, the important domains in the transmembrane region of LMP2A are targets of EBV-specific CTLs [24], [25], [26], [27], [28]. CTLs are very important in controlling the long-term persistence of EBV infection [29]. It is demonstrated that EBVaGC have much more CD8+ CTL infiltrate than EBV-negative gastric carcinoma (EBVnGC) [51], [52], and that EBVaGC generally have lower frequency of lymph node metastases and better prognosis, suggesting a protective role for the CTL infiltrate [52]. Mutation of the targets would be expected to affect the CTL response to cells expressing LMP2A and result in a growth advantage of the cells in the host [29]. In the present study, 8 of 17 CTL epitopes displayed mutations. Wang et al. [14] also reported 9 CTL epitopes mutations in LMP2A from 30 EBVaGCs in northern China, which included the 8 mutant CTL epitopes identified in the present study. The high frequency of CTL epitopes mutation may reduce or abrogate CTL responses [53], [54], and confer an advantage on EBV for immune escape and virus persistence in tumor cells, thus playing a role in tumorigenesis and tumor maintenance [29]. However, one point should be noted is that some aa changes in CTL epitopes are conservative in nature (i.c. S348T, I350V) or in non-anchoring positions and thus may not affect MHC-I binding or T-cell receptor (TCR) recognition dramatically [24]. Therefore, whether the mutations in putative CTL epitopes on LMP2A indeed affect functional recognition and thus play a role in immune selection should be confirmed in functional assays (such as IFN-γ ELISPOT assay).

Among the mutant CTL epitopes, the HLA-A11-restricted CTL epitope (codons 340 to 350) was of our great interest. The serine (S) to threonine (T) mutation at aa 348 was detected in 52.9% (9/17) of TWs of healthy EBV carriers in the present study. Tanaka *et al.* [29] and Wang *et al.* [14] also found that S or T at codon 348 was almost equally distributed in EBV isolates from healthy individuals in Japan and northern China, respectively. On the other hand, in the present study, only 6.7% (6/36) of EBVaGCs in CGC harbored this mutation (S348T). However, in northern China, S or T at codon 348 was almost equally observed in EBVaGC [14], while in Japan, the S348T mutation was predominant in EBVaGC [29]. These suggested that the polymorphisms at aa 348 may represent geographic or race-associated polymorphisms rather than disease-associated ones.

Another CTL epitope which was also of our interest is the HLA-A25-restricted CTL epitope (codons 442 to 451). In the present study, an S to T mutation at aa 444 was observed in the majority of specimens, including 80.6% (29/36) EBVaGCs in CGC, 87.5% (7/8) EBVaGCs in GRC and 88.2% (15/17) TWs of healthy EBV carriers. To date, all EBV isolates from EBVaGC in northern China (30/30) [14] and Japan (5/5) [29] harbored this mutation. Besides, all EBV isolates from NPC and healthy individuals in northern China also showed this mutation [14]. In addition to the S444T mutation which was reported previously, M443V was firstly identified in one EBVaGC case in CGC in the present study.

Another interesting finding in the present study is that the exons 5 and 8 of LMP2A were strictly conserved among EBVaGCs and TWs of healthy EBV carriers. They were also conserved in all isolates from EBVaGC in Japan (3/3) [29] and northern China (30/30) [14]. In addition, 100% (31/31) NPCs and 100% (44/44) TWs of healthy EBV carriers in northern China showed conserved sequences in exons 5 and 8 of LMP2A, too. Exon 8 of LMP2A does not contain any known CTL epitope, while exon 5 contains a HLA-A2-restricted CTL epitope (codons 329 to 337). This epitope was conserved in all isolates from eight Caucasian Hodgkin lymphoma patients and seven southern Chinese NPC patients [24]. Besides, the epitope was also conserved among EBV isolates derived from the peripheral blood of five Caucasian/African, two New Guinean and two southern Chinese donors [24]. These findings suggested that this epitope might be served as an effective target for CTL therapy for EBV-associated malignancies.

It is known that the proportion of EBVaGC in GRC was significantly higher than that in CGC [41], [42], [43], [44]. Given that, EBV seems more aggressive in EBVaGC in GRC than that in CGC. Are the LMP2A sequence variations in EBVaGC in GRC different from those in EBVaGC in CGC? In the present study, we found that in most loci of aa mutation, the number of mutations in EBVaGCs in GRC is similar to that in EBVaGCs in CGC. However, at aa codons 248, 254 and 255, which located in exon 4 of LMP2A, no mutation was detected in EBVaGCs in GRC; while aa mutation at these 3 codons were detected in 50% EBVaGCs in CGC. The difference of these mutation between EBVaGCs in GRC and EBVaGCs in CGC was statistically significant (*p* = 0.014). Besides, S348T mutation was detected in 50% EBVaGCs in GRC and 16.7% EBVaGCs in CGC. The difference of this mutation between EBVaGCs in GRC and EBVaGCs in CGC was of borderline statistically significance (*p* = 0.064). Therefore, the LMP2A sequence variations in EBVaGC in GRC seem somewhat different from those in EBVaGC in CGC. However, this may be caused by the relative small sample size in EBVaGC in GRC. Larger sample size is needed to draw a definite conclusion.

In conclusion, the present study revealed the LMP2A polymorphisms in EBVaGC in both CGC and GRC from southern China, an endemic area of NPC. The sequence variations of LMP2A in EBVaGC were similar to those in TW of healthy EBV carriers, indicating that these variations were due to geographic-associated polymorphisms rather than EBVaGC-associated mutations. The ITAM and PY motifs in the amino terminus of LMP2A were strictly conserved, suggesting their important roles in virus infection, while CTL epitopes mutations in the transmembrane region of LMP2A may implicate that the effect of LMP2A polymorphisms should be considered when LMP2A-targeted immunotherapy is conducted.
