# Correction: Sequence Variations of Latent Membrane Protein 2A in Epstein-Barr Virus-Associated Gastric Carcinomas from Guangzhou, Southern China

**DOI:** 10.1371/annotation/a68aca01-59ee-4b93-b920-af9742cf5b7c

**Published:** 2012-09-05

**Authors:** Jing Han, Jian-ning Chen, Zhi-gang Zhang, Hai-gang Li, Yun-gang Ding, Hong Du, Chun-kui Shao

There was an error in the Funding statement. The correct funding statement is: This work was supported by the National Natural Science Foundation of China (Nos. 30672409 & 81071893), the Guangdong Natural Science Foundation (Nos. 8151008901000132 & 05001748), the Guangdong Science and Technology Project (No. 2009B060700034), and the Guangzhou Science and Technology Project (No. 2011J4100106), Guangdong Province, China. The funders had no role in study design, data collection and analysis, decision to publish, or preparation of the manuscript.

